# Association between blood pressure and the risk of biopsy-induced endobronchial hemorrhage during bronchoscopy

**DOI:** 10.1186/s12890-022-01822-5

**Published:** 2022-01-06

**Authors:** Saibin Wang, Qian Ye

**Affiliations:** 1 Department of Respiratory Medicine, Jinhua Municipal Central Hospital, No. 365, East Renmin Road, Jinhua, 321000 Zhejiang Province China; 2 Department of Medical Records Quality Management, Jinhua Municipal Central Hospital, No. 365, East Renmin Road, Jinhua, 321000 Zhejiang Province China

**Keywords:** Blood pressure, Lung cancer, Bronchoscopy, Biopsy, Hemorrhage

## Abstract

**Background:**

Hemorrhage is one of the most common complications of bronchoscopy. Although several hemorrhage risk factors have been proposed, it remains unclear whether blood pressure affects the onset of biopsy-induced endobronchial hemorrhage.

**Methods:**

We conducted a retrospective cohort study of 643 consecutive adults with lung cancer over an approximately 4-year period (from January 2014 to February 2018) at a large tertiary care hospital. Patients were divided into the hemorrhage group and the non-hemorrhage group based on endobronchial biopsy (EBB) findings. The association between systolic pressure (SP), diastolic pressure (DP), mean arterial pressure (MAP), pulse pressure (PP), PP to DP ratio (PP/DP) and the risk of EBB-induced hemorrhage was evaluated using multivariate regression analysis and smooth curve fitting adjusted for potential confounding factors.

**Results:**

The EBB-induced bleeding incidence was 37.8% (243/643) in our cohort. An independent association was found between PP/PD and the EBB-induced hemorrhage risk (per 1 SD, adjusted odds ratio, 0.788; 95% confidence interval, 0.653-0.951). The multivariate regression analysis performed using quartiles of PP/DP revealed that lower level of PP/DP ratio was related to a higher risk of EBB-induced hemorrhage (*P* for trend <0.05) after adjustment for potential confounders. However, no association was observed between SP, DP, MAP, PP and EBB-induced hemorrhage.

**Conclusions:**

Low PP/DP was the independent risk factor for biopsy-induced endobronchial hemorrhage during bronchoscopy in patients with lung cancer.

## Introduction

Endobronchial biopsies (EBBs) are widely used in histopathological diagnosis of airway disorders [1]. Patients with lung cancer are the main population who is subjected to bronchoscopy and EBBs. However, hemorrhage is the most frequent and most challenging complication to handle during EBB and intraoperative hypoxemia due to increased blood loss can be life-threatening [2–4].

Several hemorrhage risk factors during EBBs have been proposed by previous studies [5, 6], including immunosuppression, mechanical ventilation, thrombocytopenia, anti-coagulant or anti-platelet therapy, severe liver and kidney disease, heart failure, pulmonary arterial hypertension, and lung transplant. Although severe hypertension (defined as systolic pressure (SP) >200 mm Hg and/or diastolic pressure (DP) >110 mm Hg) is considered a contraindication for bronchoscopy [7], it is still unclear whether blood pressure (BP) affects biopsy-induced hemorrhage.

Reportedly, arterial stiffness is an independent risk factor for anemia after percutaneous native kidney biopsy [8], and there is a positive association between arterial stiffness and BP [9]. Arterial stiffness can be evaluated using oscillometric BP measurement [10], and on the other hand, arterial stiffness measurement may be a useful tool of risk stratification in hypertension [11]. Therefore, our hypothesis was that BP might be associated with biopsy-induced hemorrhage. Hence, the objective of this study was to investigate the association between different BP components and the incidence of EBB-induced hemorrhage during bronchoscopy.

## Methods

### Study population and design

This retrospective cohort study included 643 consecutive patients who underwent EBB and were diagnosed with lung cancer over an approximately 4-year period (from January 2014 to February 2018) at the Jinhua Municipal Central Hospital. The study was approved by the Ethical Committee of the Jinhua Municipal Central Hospital (No. 2,017,101,002). The data collected from study subjects were anonymous, and informed consent was therefore waived.

Patients enrolled in this study met the following criteria: (a) adult patients with endobronchial local exophytic lesions who received forceps biopsies and (b) individuals diagnosed with a primary lung cancer. Patients experiencing the following problems were excluded from this study: immunosuppression, continuous anti-coagulant or anti-platelet therapy, severe liver and kidney disease, and active bleeding [12].

Patients were divided into two groups: patients who received hemostatic maneuvers during EBB were grouped together to form the hemorrhage group (n=243) and those who did not experience hemorrhage or did not require hemostatic maneuvers were grouped together to form the non-hemorrhage group (n=400). In addition, in this study patients were categorized into early and advanced stages based on their TNM stage (stage I and II as early stage, stage III and IV as advanced stage). Central airways referred to the trachea, left main bronchus, right main bronchus, and right middle bronchus. Peripheral bronchi referred to the left and right lobar bronchi.

### Data collection

Brachial artery pressure was measured on all patients using a sphygmomanometer (Yuyao Jiahua Medical Appliance Co., Ltd., Ningbo, China) on admission. Three BP readings were obtained from patients seated and at rest, and the mean value was used. Four BP components were collected: SP, DP, pulse pressure (PP, calculated from SP minus DP), and mean arterial pressure (MAP, calculated from [SP + (2 × DP)]/3). We also evaluated PP to DP ratio (PP/DP) to quantify pulsatility as relative PP [13].

The following covariates were collected: age, gender, body mass index (BMI), smoking history, comorbidities (chronic obstructive pulmonary disease (COPD), coronary heart disease (CHD), hypertension and diabetes), lesions location, cancer histological type and stage, biopsy hemorrhage (yes or no), and hemostatic maneuvers (4 °C 0.9% saline, diluted adrenalin (1:10,000), or/and argon plasma coagulation). The following blood parameters were measured: white blood cell (WBC) count, hemoglobin, platelets, prothrombin time (PT), activated partial thromboplastin time (APTT), C-reactive protein (CRP), triglycerides, total cholesterol (TC), high-density lipoprotein cholesterol (HDL-C), and low-density lipoprotein cholesterol (LDL-C).

### EBB procedure

EBB was performed by two experienced bronchoscopists using fiberoptic bronchoscopy (BF-1T60, Olympus Corp., Tokyo, Japan). The median time from admission to bronchoscopy was two days. Procedures were performed under general anesthesia. Propofol (Libang Pharmaceutical Co., Ltd., Xi’an, China) used for induction (1.0 mg/kg) and for maintenance (3.0-6.0 mg/kg/h), and remifentanil (Jiangsu Hengrui Medicine Co., Ltd., Lianyungang, China) (5.0-10.0 µg/kg/h) used for sedation and analgesia. Patients were intubated with a laryngeal mask airway (Well Lead Medical Co., Ltd., Guangzhou, China), and ventilated using a closed circuit connected to the ventilator (Fabius Tiro (Draeger Medical GmbH, Luebeck, Germany). Bronchoscopic procedures were performed when patients were under laryngeal mask airway. Biopsy forceps (outer diameter 1.8mm) (QYQ-FC 1.8 × 1100, Jinlong Medical Plastic Instrument Co., Ltd., Changzhou, China) were used during bronchoscopy. Three to five biopsies were performed on each patient at the same lesion location [1], but only one biopsy was performed when some lesions bled significantly following the first biopsy attempt. During bronchoscopy, 4 °C 0.9% saline, diluted adrenalin (1:10,000), and argon plasma coagulation were used for hemostasis, if needed.

### Statistical analysis

All analyses were performed using R software (The R Foundation; http://www.Rproject. org) and Empower software (X&Y solutions, Inc., Boston, MA; http://www.empowerstats. com). Descriptive statistics were used to summarize baseline characteristics. Continuous variables were presented as median with interquartile range (IQR), and categorical variables were expressed as the number (percentage). Data were also expressed as odds ratio (OR) and 95% confidence interval (CI). PP/PD measurements were also presented as Z-scores in multivariate regressions. Between-group comparisons were executed using unpaired *t*-tests (normal distribution) or Kruskal-Wallis rank sum test (non-normal distribution), Pearson chi-squared tests or the Fisher’s exact, as appropriate. Multivariate logistic regression and generalized additive model with smooth curve fitting were conducted for analyzing the association between different BP components and risk of EBB-induced hemorrhage, with an adjustment for potential confounders. The adjusted criteria I included risk factors producing a change in the regression coefficient greater than 10% after introduction into the basic model (age, location of lesion, and histological types), or the regression coefficient of co-variable to dependent variable of *P* < 0.1 (smoking, CRP, PT, APTT, HDL-C, and triglycerides), while the screening criteria II included risk factors judged by clinical significance (stage of cancer, BMI, WBC, and platelets). A two-tailed *P* value< 0.05 was considered statistically significant.

## Results

Among the 643 patients, 243 (37.8%) experienced EBB-associated hemorrhage. These patients received hemostatic treatment. However, severe hemorrhage (defined as a single amount of biopsy-induced blood loss ≥100ml) was not observed in any patient. Patient’s demographics and blood tests are shown in Table 1.

**Table 1:** Baseline characteristics and blood tests of study patients

Variables	Non-hemorrhaging	Hemorrhaging	*P *value
	n = 400	n = 243	
Baseline characteristics			
Age, year, median (IQR)	65 (59, 70)	65 (60, 71)	0.146
BMI, kg/m^2^, median (IQR)	21.8 (19.7, 24.0)	21.5 (19.7, 23.8)	0.308
Gender, n (%)			0.133
Female	96 (24.00)	46 (18.93)	
Male	304 (76.00)	197 (81.07)	
Smoking, n (%)			0.020
Never	163 (40.75)	76 (31.28)	
Former	55 (13.75)	49 (20.16)	
Current	182 (45.50)	118 (48.56)	
Location of lesion, n (%)			<0.001
Peripheral bronchi	31 (7.75)	51 (20.99)	
Central airway	369 (92.25)	192 (79.01)	
Stage, n (%)			<0.001
Early	241 (60.25)	108 (44.44)	
Advanced	159 (39.75)	135 (55.56)	
Histological types, n (%)			<0.001
Adenocarcinoma	133 (33.25)	40 (16.46)	
Squamous cell carcinoma	177 (44.25)	145 (59.67)	
SCLC	69 (17.25)	45 (18.52)	
Others	21 (5.25)	13 (5.35)	
Hypertension, n (%)			0.719
No	303 (75.75)	181 (74.49)	
Yes	97 (24.25)	62 (25.51)	
Diabetes, n (%)			0.683
No	379 (94.75)	232 (95.47)	
Yes	21 (5.25)	11 (4.53)	
CHD, n (%)			0.626
No	388 (97.00)	234 (96.30)	
Yes	12 (3.00)	9 (3.70)	
COPD, n (%)			0.967
No	374 (93.50)	227 (93.42)	
Yes	26 (6.50)	16 (6.58)	
Different components of blood pressure, mmHg, median (IQR)			
SP	131 (119, 144)	130 (116, 145)	0.173
DP	77 (70, 85)	78 (70, 88)	0.331
MAP	96 (88, 104)	95 (86, 106)	0.920
PP	53 (43, 64)	52 (40, 61)	0.015
PP/DP, median (IQR)	0.68 (0.54, 0.84)	0.65 (0.50, 0.79)	0.012
Laboratory values, median (IQR)			
WBC (×10^9^/L)	6.7 (5.4, 8.4)	6.9 (5.7, 8.8)	0.155
Hemoglobin (g/dL)	12.8 (11.6, 13.9)	12.8 (11.4, 14.0)	0.403
Platelets (×10^9^/L)	220 (171, 278)	231 (173, 287)	0.256
CRP (mg/L)	6.7 (1.1, 24.7)	10.7 (2.4, 40.2)	0.011
PT (S)	13.1 (12.5, 13.7)	12.7 (11.6, 13.4)	0.063
APTT (S)	35.4 (32.5, 38.8)	33.9 (30.7, 37.6)	0.006
Triglycerides (mmol/L)	1.1 (0.8, 1.5)	1.0 (0.8, 1.3)	0.079
TC (mmol/L)	4.1 (3.5, 4.8)	4.1 (3.5, 4.7)	0.375
HDL-C (mmol/L)	1.2 (1.0, 1.4)	1.1 (0.9, 1.3)	0.026
LDL-C (mmol/L)	2.8 (2.3, 3.3)	2.8 (2.3, 3.2)	0.290

BMI, body mass index; SP, systolic pressure; DP, diastolic pressure; MAP, mean arterial pressure; PP, pulse pressure; PP/DP, pulse pressure to diastolic pressure ratio; SCLC, small-cell lung carcinoma; CHD, coronary heart disease; COPD, chronic obstructive pulmonary disease; WBC, white blood cell; CRP, C-reactive protein; PT, prothrombin time; APTT, activated partial thromboplastin time; TC, total cholesterol; HDL-C, high-density lipoprotein cholesterol; LDL-C, low-density lipoprotein cholesterol

**Fig. 1:**
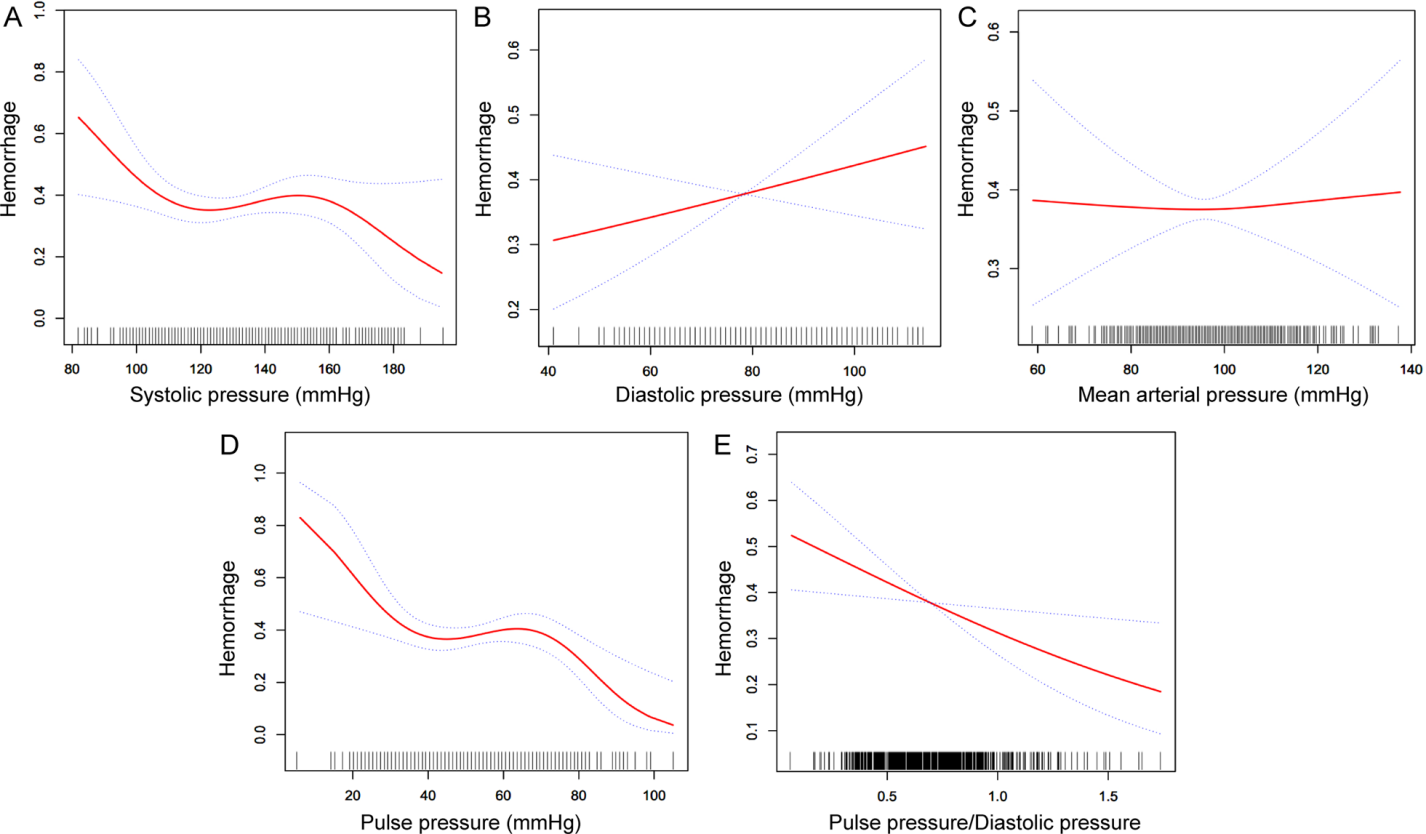
The smooth curve fitting shows the association between the risk of endobronchial biopsy-induced hemorrhage and systolic pressure (**a**), diastolic pressure (**b**), mean arterial pressure (**c**), pulse pressure (**d**), and pulse pressure to diastolic pressure ratio (**e**) after adjustment for age, gender, BMI, smoking, lesion locations, cancer histological type and stage, PT, APTT, triglycerides, HDL-C, WBC, platelets, and CRP. Dotted lines represented the upper and lower 95% confidence intervals. BMI, body mass index; PT, prothrombin time; APTT, activated partial thromboplastin time; HDL-C, high-density lipoprotein cholesterol; WBC, white blood cell; CRP, C-reactive protein

The associations between SP, DP, MAP and EBB-induced hemorrhage risk were not statistically significant before or after adjustment for potential confounders in multivariate regression analysis (Table 2, *P* > 0.05). The relationships between SP, DP, MAP and EBB-induced hemorrhage risk in smooth curve fitting are shown in Fig. 1a–c after adjustment for potential confounders.

**Table 2:** Multivariate regression analysis of the peripheral blood pressure with risk of endobronchial biopsy-induced hemorrhage

Components of blood pressure (mmHg)	n	Odds ratio (95% CI)	
		Non-adjusted	Adjusted
Systolic blood pressure			
Q1 (82-117)	159	1.0	1.0
Q2 (118-130)	159	0.688 (0.437, 1.084)	0.710 (0.435, 1.158)
Q3 (131-144)	164	0.772 (0.494, 1.207)	0.814 (0.493, 1.345)
Q4 (145-195)	161	0.816 (0.522, 1.277)	0.806 (0.482, 1.347)
P for trend		0.460	0.500
Continuous, per 10 mmHg		0.994 (0.986, 1.002)	0.993 (0.984, 1.002)
Diastolic blood pressure			
Q1 (41-69)	154	1.0	1.0
Q2 (70-77)	164	0.932 (0.589, 1.476)	1.019 (0.622, 1.670)
Q3 (78-86)	162	1.029 (0.651, 1.627)	0.990 (0.600, 1.636)
Q4 (87-114)	163	1.317 (0.839, 2.069)	1.452 (0.870, 2.426)
P for trend		0.179	0.156
Continuous, per 10 mmHg		1.007 (0.993, 1.020)	1.007 (0.991, 1.022)
Mean arterial pressure			
Q1 (59-87)	158	1.0	1.0
Q2 (88-95)	161	0.785 (0.500, 1.232)	0.870 (0.536, 1.412)
Q3 (96-104)	161	0.703 (0.446, 1.109)	0.697 (0.423, 1.150)
Q4 (104-138)	163	0.925 (0.592, 1.443)	0.895 (0.540, 1.485)
P for trend		0.690	0.553
Continuous, per 10 mmHg		0.999 (0.987, 1.012)	0.998 (0.984, 1.012)

*Adjusted for age, gender, BMI, smoking, lesion locations, cancer histological type and stage, PT, APTT, triglycerides, HDL-C, WBC, platelets, and CRP

BMI, body mass index; PT, prothrombin time; APTT, activated partial thromboplastin time; HDL-C, high-density lipoprotein cholesterol; WBC, white blood cell; CRP, C-reactive protein

In unadjusted models, a strong significant association was observed between PP/DP and EBB-induced hemorrhage risk. Table 3 shows that every 1 SD (1 SD = 0.24 PP/DP) increase of PP/DP, the odds of hemorrhage decreased by about 19.0%. After additional adjustment for potential confounders (age, gender, BMI, smoking, lesion locations, cancer histological type and stage, PT, APTT, triglycerides, HDL-C, WBC, platelets, and CRP), the strength of this association remained significant. It indicates that every 1 SD increase of PP/DP, the risk of biopsy-induced endobronchial hemorrhage decreased by about 21.2% (Table 3). Moreover, in the multivariate regression analysis using quartiles of PP/PD, whether or not the potential confounders were adjusted, a trend of an increased incidence of EBB-induced hemorrhage was observed in patients with a lower PP/DP level (Table 3, *P* for trend < 0.05). Although 10 mm increase in PP contributed to a decrease in the hemorrhage risk after adjustment for the aforementioned factors (Table 3, OR, 0.986; 95% CI, 0.974-0.997), in the multivariate regression analysis using quartiles of PP, however, no variation tendency was observed between higher PP and EBB-induced hemorrhage risk (Table 3, *P* for trend > 0.05). The association between PP, PP/PD ratio and EBB-induced hemorrhage risk is shown in Fig. 1 d, e after adjustment for potential confounders.

**Table 3:** Multivariate regression analysis of pulsatility with the risk of endobronchial biopsy-induced hemorrhage

Pulsatility	n	Odds ratio (95% CI)	
		Non-adjusted	Adjusted*
Pulse pressure (mmHg)			
Q1 (6-41)	157	1.0	1.0
Q2 (42-51)	149	0.807 (0.510, 1.277)	0.770 (0.469, 1.265)
Q3 (52-62)	173	0.915 (0.590, 1.419)	0.932 (0.570, 1.524)
Q4 (63-105)	164	0.658 (0.417, 1.038)	0.606 (0.361, 1.017)
P for trend		0.113	0.101
Continuous, per 10 mmHg		0.987 (0.977, 0.998)	0.986 (0.974, 0.997)
PP/DP			
Q1 (0.07-0.52)	160	1.0	1.0
Q2 (0.53-0.66)	161	0.653 (0.417, 1.023)	0.652 (0.400, 1.064)
Q3 (0.67-0.83)	161	0.727 (0.466, 1.135)	0.729 (0.445, 1.193)
Q4 (0.84-1.74)	161	0.569 (0.361, 0.895)	0.533 (0.319, 0.891)
P for trend		0.028	0.029
**Continuous, per SD		0.810 (0.685, 0.956)	0.788 (0.653, 0.951)

*Adjusted for age, gender, BMI, smoking, lesion locations, cancer histological type and stage, PT, APTT, triglycerides, HDL-C, WBC, platelets, and CRP

BMI, body mass index; PT, prothrombin time; APTT, activated partial thromboplastin time; HDL-C, high-density lipoprotein cholesterol; WBC, white blood cell; CRP, C-reactive protein; PP, pulse pressure; DP, diastolic pressure; SD, standard deviation

**1 SD=0.24 PP/DP

## Discussion

In the present study, we found that PP/DP ratio was associated with EBB-induced hemorrhage risk during bronchoscopy in patients with lung cancer. Lower PP/DP ratio was the independent risk factor for EBB hemorrhage. Since few studies focused on the association between different BP components and risk of EBB-induced hemorrhage, our findings are novel and might indicate that BP is a modifiable factor to reduce EBB-induced hemorrhage.

Hemorrhage is the most common and clinically relevant complication after endobronchial biopsy, and it is perhaps the most distressing and challenging to manage for bronchoscopists [2], especially when a massive hemorrhage occurs. In the non-selective study population, bronchoscopy-induced hemorrhage was reported between 1% and 20% [2]. However, the incidence of hemorrhage increases significantly in subjects that need a biopsy [14]. In addition, malignant lesions are more likely subjected to bleed upon biopsy than benign mucosal lesions [15]. The rate of EBB-induced hemorrhage can even reach 30.5% in patients with lung cancer [16].

Despite the controversy and insufficient evidence [17, 18], several risk factors for hemorrhage during bronchoscopy have been proposed, including immunosuppression, mechanical ventilation, thrombocytopenia (platelets < 50 × 10^3^/µL), anticoagulant and antiplatelet drug use, heart failure, liver and kidney disease, lung transplant, and bleeding tendencies [5, 6]. However, to the best of our knowledge, among these factors no feasible and effective indicators to predict EBB-induced hemorrhage are available in clinical practice.

Several studies have shown that there was a strong possibility of a relationship between BP and biopsy-related bleeding. Reportedly, increased PP may be a cause of the high bleeding rate in percutaneous kidney biopsy, and treatments that can reverse arterial stiffness may reduce the possibility of renal biopsy-induced bleeding [8]. In addition, in several retrospective studies, indices from SP and DP measurements resulted valid means of detecting blood loss, and showed good sensitivity and specificity in predicting large blood losses [19, 20]. Although BP may play a role in some hemorrhagic diseases, there is a paucity of data to evaluate BP in EBB. To understand better the risk factors of hemorrhage during EBB, we explored the relationship between BP and biopsy hemorrhage.

The main result of our study was that PP/DP ratio was associated with EBB-induced hemorrhage risk during bronchoscopy in patients with lung cancer. Because PP absolute values were affected by changes in DP [21], it should be normalized by the use of reasonable values. Therefore, we used the PP/DP ratio [13]. Decreased PP/PD ratio was independently associated with increased risk of hemorrhage during EBB. Even after adjustment for potential confounders (age, gender, BMI, smoking, lesion locations, cancer histological type and stage, PT, APTT, triglycerides, HDL-C, WBC, platelets, and CRP), the association was significant. Despite the mechanism underlying the relationship between PP/PD ratio and the risk of biopsy-induced hemorrhage remains unknown; we think that this parameter, with noninvasive characteristics, easily available and almost no expensive, might have the potential to become a clinical reference indicator.

The strengths of the present study included inclusion of consecutive patients, experienced bronchoscopists for EBB procedure, using only one biopsy method (EBB), and a relatively fixed number of biopsies, as well as a uniform method of BP measurement. However, several limitations are present in the current study. First, the retrospective nature of the study implies that several potential confounders might not have been considered and some complications might be underestimated. Thus, further validation in prospective studies should be considered. Second, this study was performed at a single center and all participants came from East China, therefore, it is unknown whether the results could be valid for other medical facilities. Moreover, the use of general anesthesia and laryngeal mask is the standard of care for EBB at our institution, and in this case facilitates the successful implementation of intraoperative biopsy. However, this also suggests that the results of this study may not be extrapolated to the risk assessment of biopsy bleeding in patients who underwent EBB without general anesthesia and laryngeal mask. Third, BP on admission was measured, regardless of whether the patient took antihypertensive drugs or not. In addition, at present, an accurate measure of the blood loss amount during bronchoscopy is difficult to be evaluated [22]. A quantitative measurement of the volume of EBB-induced hemorrhage could not be provided in our study. We categorized patients into the hemorrhage group or non-hemorrhage group just based on whether they received hemostasis treatment following EBB. Therefore, for some patients with minimal bleeding, this classification may fail to achieve accurate grouping. Despite some potential limitations, our study was the first revealing that PP/PD ratio was associated with EBB-induced hemorrhage risk and it may facilitate the bronchoscopist to assess the risk of intraoperative bleeding.

## Conclusions

There was no significant correlation between SP, DP, MAP, PP and the risk of EBB-induced endobronchial hemorrhage during bronchoscopy in patients with lung cancer. However, low PP/PD ratio was associated with an increased EBB-induced hemorrhage risk. Thus, PP/PD ratio has the potential to be used for risk assessment and risk modification prior to bronchoscopy.

## Data Availability

The datasets used and/or analysed during the current study are available from the corresponding author on reasonable request.

## Abbreviations

EBB: endobronchial biopsy
BMI: body mass index
SP: systolic pressure
DP: diastolic pressure
MAP: mean arterial pressure
PP: pulse pressure
PP/DP: pulse pressure to diastolic pressure ratio
SCLC: small-cell lung carcinoma
CHD: coronary heart disease
COPD: chronic obstructive pulmonary disease
WBC: white blood cell
CRP: C-reactive protein
PT: prothrombin time
APTT: activated partial thromboplastin time
TC: total cholesterol
HDLC: high-density lipoprotein cholesterol
LDLC: low-density lipoprotein cholesterol

## References

[1] Rivera MP, Detterbeck F, Mehta AC (2017). Management of antithrombotic agents in patients undergoing flexible bronchoscopy. Eur Respir Rev.

[2] Cordasco EM, Mehta AC, Ahmad M (1991). Bronchoscopically induced bleeding-a summary of nine years’ Cleveland clinic experience and review of the literature. Chest.

[3] Herth FJF (2017). Bronchoscopy and bleeding risk. Eur Respir Rev.

[4] Chinsky K (2005). Bleeding risk and bronchoscopy: in search of the evidence in evidence-based medicine. Chest.

[5] Herth FJ, Becker HD, Ern A (2002). Aspirin does not increase bleeding complications after transbronchial biopsy. Chest.

[6] Diette GB, Wiener CM, White P (1999). The higher risk of bleeding in lung transplant recipients from bronchoscopy is independent of traditional bleeding risks: results of a prospective cohort study. Chest.

[7] Van Gundy K, Boylen CT (1988). Fiberoptic bronchoscopy. Indications, complications, contraindications. Postgrad Med.

[8] Tanaka K, Kitagawa M, Onishi A, Yamanari T, Ogawa-Akiyama A, Mise K, Inoue T, Morinaga H, Uchida HA, Sugiyama H, Wada J (2017). Arterial Stiffness is an Independent Risk Factor for Anemia After Percutaneous Native Kidney Biopsy. Kidney Blood Press Res.

[9] Wen W, Luo R, Tang X, Tang L, Huang HX, Wen X, Hu S, Peng B (2015). Age-related progression of arterial stiffness and its elevated positive association with blood pressure in healthy people. Atherosclerosis.

[10] Komine H, Asai Y, Yokoi T, Yoshizawa M (2012). Non-invasive assessment of arterial stiffness using oscillometric blood pressure measurement. Biomed Eng Online.

[11] Nemcsik J, Cseprekál O, Tislér A (2017). Measurement of Arterial Stiffness: A Novel Tool of Risk Stratification in Hypertension. Adv Exp Med Biol.

[12] Miller RJ, Casal RF, Lazarus DR, Ost DE, Eapen GA (2018). Flexible Bronchoscopy. Clin Chest Med.

[13] Sunagawa K, Burkhoff D, Lim KO, Sagawa K (1982). Impedance loading servo pump system for excised canine ventricle. Am J Physiol.

[14] Zavala DC (1976). Pulmonary hemorrhage in fberoptic transbronchial biopsy. Chest.

[15] Ozgül MA, Turna A, Yildiz P, Ertan E, Kahraman S, Yilmaz V (2006). Risk factors and recurrence patterns in 203 patients with hemoptysis. Tuberk Toraks.

[16] Wang S, Ye Q, Tu J, Song Y (2018). The location, histological type and stage of lung cancer are associated with bleeding during endobronchial biopsy. Cancer management and research.

[17] Carr IM, Koegelenberg CF, von Groote-Bidlingmaier F, Mowlana A, Silos K, Haverman T, Diacon AH, Bolliger CT (2012). Blood loss during flexible bronchoscopy: a prospective observational study. Respiration.

[18] Zahreddine I, Atassi K, Fuhrman C (2003). Impact of prior biological assessment of coagulation on the hemorrhagic risk of fiberoptic bronchoscopy. Rev Mal Respir.

[19] Ardagh MW, Hodgson T, Shaw L, Turner D (2001). Pulse rate over pressure evaluation (ROPE) is useful in the assessment of compensated hemorrhagic shock. Emerg Med (Fremantle).

[20] King RW, Plewa MC, Buderer NM, Knotts FB (1996). Shock index as a marker for significant injury in trauma patients. Acad Emerg Med.

[21] Yamashita N, Nakayama Y, Tsumura K, Nishijima T, Ueda H, Yoshimaru K, Hayashi T, Yoshikawa J (2001). Pulsatility of brachial artery pressure is associated with an increased risk of coronary artery disease in men. Journal of Hypertension.

[22] Schumann C, Hetzel M, Babiak AJ, Hetzel J, Merk T, Wibmer T, Lepper PM, Krüger S (2010). Endobronchial tumor debulking with a flexible cryoprobe for immediate treatment of malignant stenosis. J Thorac Cardiovasc Surg.

